# Incorporation of Diketopyrrolopyrrole into Polythiophene for the Preparation of Organic Polymer Transistors

**DOI:** 10.3390/molecules29010260

**Published:** 2024-01-03

**Authors:** Shiwei Ren, Zhuoer Wang, Wenqing Zhang, Abderrahim Yassar, Jinyang Chen, Sichun Wang

**Affiliations:** 1 Zhuhai-Fudan Research Institute of Innovation, Hengqin 519000, China; shiwei_ren@fudan.edu.cn; 2 Zhejiang Key Laboratory of Alternative Technologies for Fine Chemicals Process, Shaoxing University, Shaoxing 312000, China; 3 Department of Materials Science, Fudan University, Shanghai 200438, China; 4 Key Laboratory of Colloid and Interface Chemistry of Chemistry and Chemical Engineering, Shandong University, Jinan 250100, China; 5 Key Laboratory of Organic Solids, Institute of Chemistry, Chinese Academy of Sciences, Beijing 100190, China; zhangwq@iccas.ac.cn; 6 Laboratory of Physics of Interfaces and Thin Films, Institut Polytechnique de Paris, 91128 Palaiseau, France; abderrahim.yassar@polytechnique.edu

**Keywords:** polythiophene, organic electronics, solution processing, π-conjugated materials, transistor, hole mobility

## Abstract

Polythiophene, as a class of potential electron donor units, is widely used in organic electronics such as transistors. In this work, a novel polymeric material, PDPPTT-FT, was prepared by incorporating the electron acceptor unit into the polythiophene system. The incorporation of the DPP molecule assists in improving the solubility of the material and provides a convenient method for the preparation of field effect transistors via subsequent solution processing. The introduction of fluorine atoms forms a good intramolecular conformational lock, and theoretical calculations show that the structure displays excellent co-planarity and regularity. Grazing incidence wide-angle X-ray (GIWAXS) results indicate that the PDPPTT-FT is highly crystalline, which facilitates carrier migration within and between polymer chains. The hole mobility of this π-conjugated material is as high as 0.30 cm^2^ V^−1^ s^−1^ in organic transistor measurements, demonstrating the great potential of this polymer material in the field of optoelectronics.

## 1. Introduction

**Figure 1:**
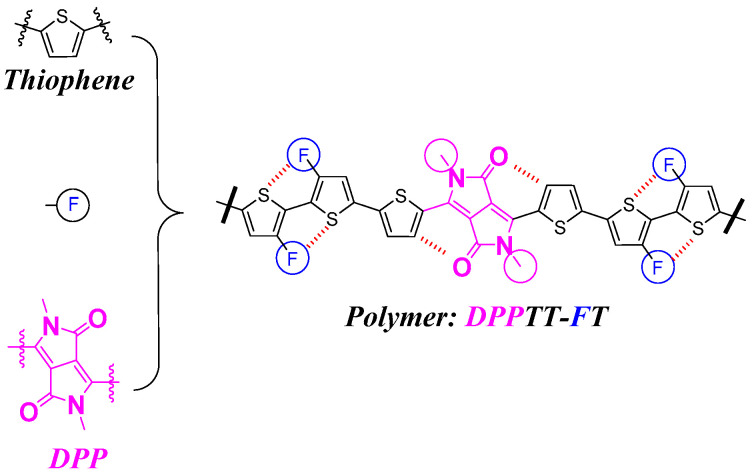
Molecular structure design strategies for target compound with polythiophene units.

The development of π-conjugated polymer semiconductors and polymer field effect transistors (PFETs) has been a great achievement in the last decade [1,2,3]. Polymer semiconductor materials, as the core component of organic field effect transistors (OFETs), are mainly distinguished from devices based on small-molecule materials [4,5,6,7]. In general, polymer semiconductor materials are distinguished by their solution processability and film-forming properties. The widely used donor–acceptor (D–A) strategy has played a great role in this dynamic development, which is mainly related to the flexibility to modulate the energy levels of the materials and the significant increase in the diversity of the molecular structure library [8,9,10]. Polymers and oligomers of conjugated thiophene have received much attention because of their strong electron-donating ability and are excellent donor units [11,12,13]. The earliest studies on the synthesis of polythiophene and its application in transistors date back to 1980 and 1986, respectively. One of the reasons for the low carrier mobility is mainly due to the inferior solubility of the material limiting its processability [14]. Considering that the thiophene unit contains unsubstituted positions, the appropriate introduction of solubilizing groups, such as alkyl chains, alkoxy chains, etc., is assumed to be an effective way to significantly enhance the solubility of the material. A typical example of molecular structures is poly(3-hexylthiophene), (P3Ht), which has been developed and utilized in the field of transistors. Bielecka and Jiang reported that this type of material shows moderate mobility in the range of 10^−4^ to 10^−2^ cm^2^ V^−1^ s^−1^ [15,16]. Similarly, Shao et al. reported the introduction of hydrophilic chains to prepare P3TEGT, which is an environmentally friendly transistor material with high solubility [17]. One of the ways to further improve the performance of such material devices is to reduce the number of regioisomers in the material system, that is, to control the conformation of regioirregular isomers connected via three types of linkages: head-to-tail, head-to-tail and tail-to-tail [18]. On the other hand, direct linkage with solubilizing groups, which does not affect the conjugated structure of the material and increases the number of isomers, has been considered as a straightforward and convenient means to improve the solubility and processability of the material. Herein, we aim to further introduce diketopyrrolopyrrole (DPP) units on the polythiophene chain, which improves the solubility of the material through its side chains, both of which contain long alkyl chains [19]. More importantly, DPP itself is an electron acceptor unit, which can further significantly improve the carrier mobility and device performance of the material through the donor–acceptor interactions within the material [20,21]. The introduction of the fluorine atom serves two purposes. Firstly, an intramolecular conformational lock is formed through the F…S non-covalent bonding interaction, which improves the regularity of the material [22]. The second is to lower the frontier molecular orbital energy level (FMO) of the material to further improve the device performance [23,24]. The molecular structure is designed as shown in Figure 1 below, where a tiny fraction of DPP along with polythiophene units are used to prepare polymers via Stille coupling and to further explore its properties in the field of organic transistors.

## 2. Results

### 2.1. Synthesis and Characterization of π-Conjugated Material PDPPTT-FT

**Scheme 1:**
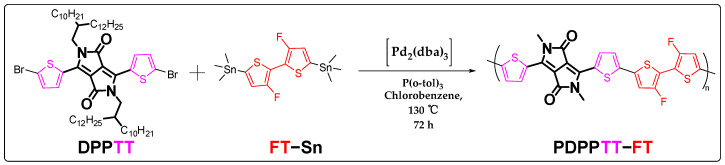
Synthesis conditions and routes for the π-conjugated polymer PDPP-5Th.

**Figure 2:**
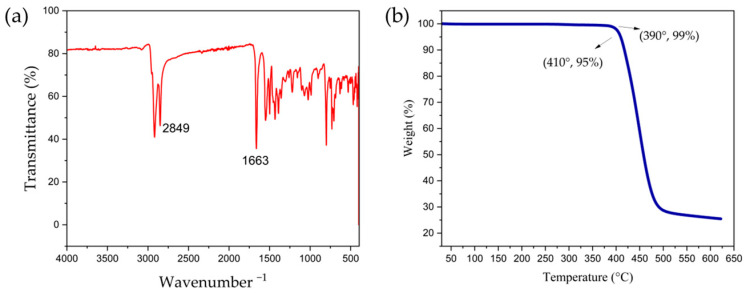
(**a**) IR spectrum and (**b**) thermal decomposition curve of PDPPTT-FT.

The preparation of the polymer PDPPTT-FT relies on the palladium-catalyzed coupling polymerization of 3,6-bis(5-bromothiophen-2-yl)-2,5-bis(2-decyltetradecyl)-2,5-dihydropyrrolo [3,4-c]pyrrole-1,4-dione (DPPTT) with monomer [4-fluoro-5-(3-fluoro-5-trimethylstannylthiophen-2-yl)thiophen-2-yl]-trimethylstannane (FT-Sn), as shown in Scheme 1. The specific synthetic route is described in the Materials and Methods section. The DPPTT moiety contains a DPP structural unit, which is directly attached to the thiophene ring (TT) by a single bond on both sides, and has two long non-conjugated alkyl chains in the side chain. DPP is an excellent electron acceptor unit with a long alternating single- and double-bond π-conjugated structure and the carbonyl functional group. The extremely long aliphatic chain as the side chain facilitates the solubility and processability of the material in organic solvents. Meanwhile, monomer FT-Sn contains double trimethyltin functional groups and can easily undergo a Stille coupling reaction with monomer DPPTT [25]. The crude product obtained can be further purified via Soxhlet extraction to remove impurities such as oligomers or catalyst ligands [26]. Polymeric target materials with suitable conjugation lengths show excellent solubility in chlorinated solvents such as dichloromethane, chloroform, or chlorobenzene. Figure 2a shows the infrared (IR) spectrum of the polymer with its typical carbonyl and alkyl chain peaks appearing near 2849 cm^−1^ and 1663 cm^−1^, respectively (Supplementary Materials). Based on high-temperature gel permeation chromatography, the values of the average molecular weight (Mn) and weight average of the molecular weight (Mw) of the polymer were shown to be 23.6 k and 88.1 k, respectively, which corresponds to a polydispersity index (PDI) of 3.73 (Figure S1). The average minimum repeating units contained in the polymer chains are 20 and 75 based on Mn and Mw speculation. The results of the elemental analyses are shown in Table 1. The experimental values for the three elements are close to the theoretical values, with an overall variation of around 0.5% [27,28]. Figure 2b below shows the thermodynamic decomposition temperature curve of the material, which was chosen for the annealing temperature in subsequent device preparations. The material shows excellent stability up to 390 °C of heating. Its mass loss at 5% is found to be around 410 °C, and subsequent heating to 450 °C causes a significant decomposition of the material.

### 2.2. Density Functional Theory (DFT) Calculation

**Figure 3:**
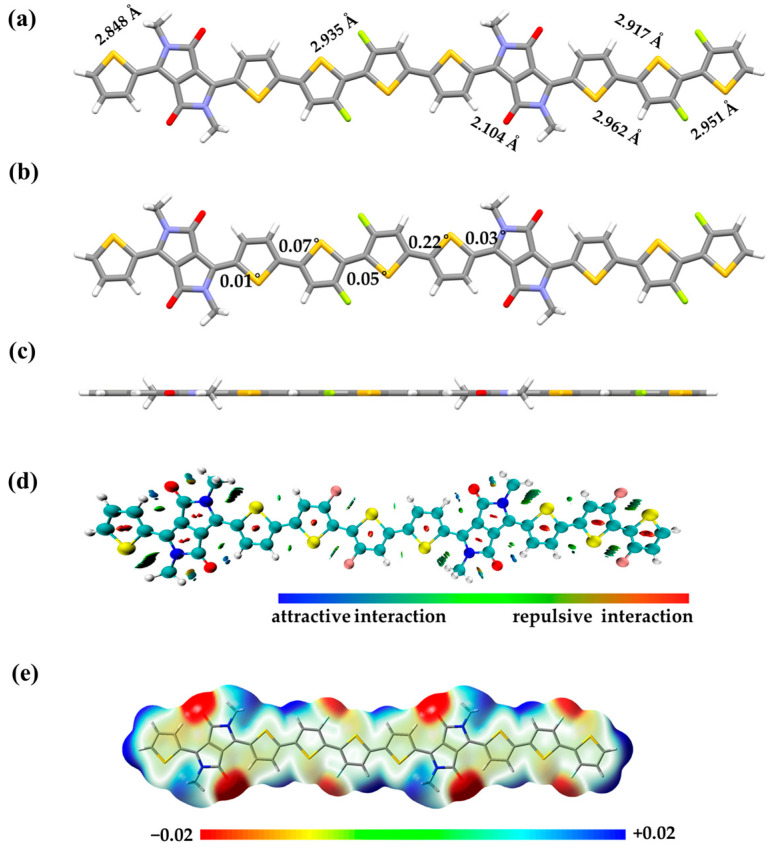
(**a**) Intramolecular distances, (**b**) dihedral angles and (**c**) side view of the dimer; (**d**) NCI and (**e**) ESP analysis of the methyl-substituted dimer.

To further characterize the intramolecular conformation of PDPPTT-FT, DFT calculations were used based on the B3LYP-D3/6-311G(d,p) level [29,30] of Gaussian 16 [31]. Dispersion was introduced to further obtain accurate conformational information to study non-covalent interactions [32]. Methyl chain-substituted dimers were taken as models for the calculations in order to simplify and reduce the computation time [33,34,35]. Typical intramolecular atomic distances and dihedral angles between fragments are shown in Figure 3a,b, respectively. The DPP unit consists of two five-membered fused rings with good coplanarity. The distance between the oxygen atom in the carbonyl group and the hydrogen atom on the neighboring thiophene is only 2.1 Å, which is smaller than the van der Waals force radius and favors the formation of intramolecular hydrogen bonds. The distance between the fluorine (F) atom and the sulfur (S) atom of the neighboring thiophene is 2.9 Å, which facilitates the formation of intramolecular conformational locks and further stabilizes the conformation of the material. The thiophenes are arranged alternately and with small dihedral angles, which results in an overall excellent planarity of the material (Figure 3c). Most of the dihedral angles are in the range of 2°, which is more coplanar compared to systems based on polythiophene or fluorinated polythiophene [36]. The high planarity enables the increase in the effective conjugation length and the formation of fine stacking, which in turn facilitates the efficient carrier migration. Intramolecular hydrogen bonding and F...S interactions are further verified through the weak force analysis in Figure 3d. Intramolecular non-covalent bond interaction (NCI) analyses showed a distinct blue–green coloration at their corresponding positions, illustrating these attractive interactions [37]. Based on the electrostatic surface potential (ESP) analysis of the molecule in Figure 3e, it is shown that the material displays an electron-donating character as a whole, with a strong electron-withdrawal effect only around its carbonyl groups and fluorine atoms. The above results suggest that the material has a stronger hole transport capacity as compared to the electron transport capacity.

**Figure 4:**
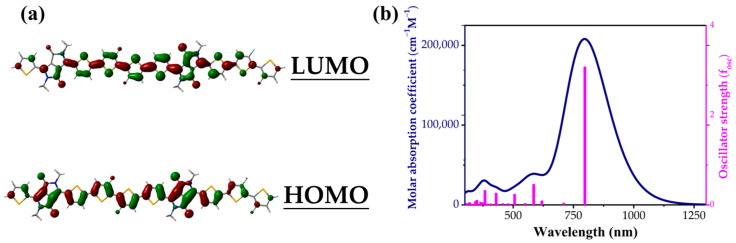
(**a**) The HOMO and LUMO orbital energy diagram; (**b**) the simulated UV–Vis spectra of methyl-substituted dimer of PDPPTT-FT.

Next, we calculated the highest occupied molecular orbital (HOMO) and the lowest unoccupied molecular orbital (LUMO) energy levels of the π-conjugated material, which resulted in −5.27 eV and −3.56 eV, respectively, as shown in Figure 4a. The corresponding calculated results for HOMO−1 and HOMO−2 are shown in Figure S2. Figure 4b simulates the ultraviolet–visible (UV–Vis) absorption, and the material exhibits strong absorption in the range of 350–1150 nm. Absorption based on the range from 750 nm to 950 nm is due to intramolecular charge transfer, while high-energy absorption between 300 nm and 600 nm is relatively insignificant. The main and onset absorption peaks of the material are at 797 nm and 1020 nm, respectively. The material has a strong molar absorption coefficient and a large oscillator strength (f_osc_) of 207.2 k and 3.06, respectively, which are due to the good linear conjugate arrangement within the molecular structure [38]. The spatial atomic coordinates of the structure are summarized in Table S1.

### 2.3. Electrochemical Properties

**Figure 5:**
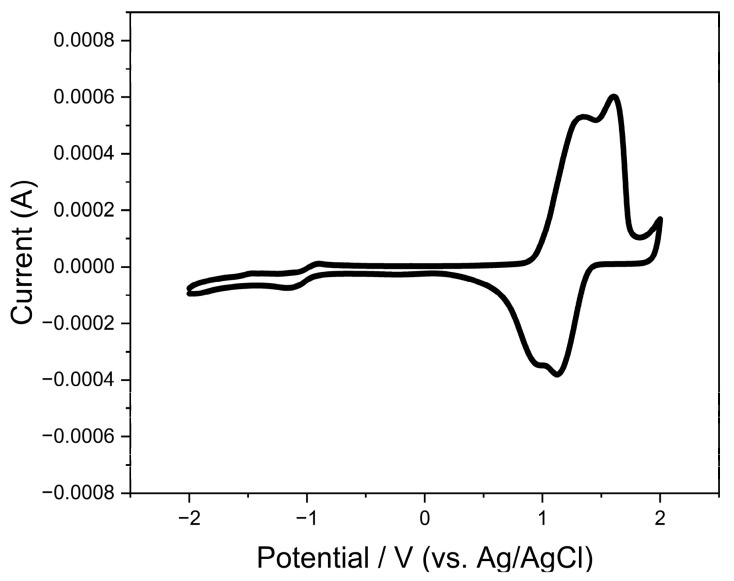
Redox characteristic of π-conjugated polymer PDPPTT-FT (scanning rate: 0.1 V/s).

We have performed electrochemical tests on the films with cyclic voltammetry measurements to study the redox properties and the frontier molecular orbital energy levels of the material, as shown in Figure 5 below. The higher peak intensity of the oxidation properties of the material compared to the reduction properties suggests that the potential for hole transport should be superior to that of electron transport. Specifically, the polymer material reveals well-reversible double oxidation peaks at 1.35 V and 1.62 V, respectively. On the other hand, the material exhibits a well-defined mono-reversible reduction feature, with the reduction peak arising at −1.12 V. According to the onset of the oxidation peak and the onset of the reduction peak (0.90 V of E_ox_^onset^ and −0.94 V of E_red_^onset^, respectively), we can deduce the corresponding HOMO and LUMO energy levels of the material to be −5.30 eV and −3.46 eV, respectively. The specific formula for the energy level calculation is: E_LUMO_ = −4.80 eV − [(E_red_^onset^) − E_1/2_(ferrocene)] and E_HOMO_ = −4.80 eV − [(E_ox_^onset^) − E_1/2_(ferrocene)], where E_1/2_(ferrocene) = 0.40 eV. The LUMO energy level of PDPPTT-FT is significantly lower compared to that of polythiophene, which is directly related to the insertion of the acceptor moiety. On the other hand, its energy level is closer to that of fluorinated polythiophene [39]. It is worth mentioning that the HOMO energy levels obtained from electrochemical tests are close to those calculated from theoretical simulations. The theoretical values of the LUMO energy levels are slightly different from those in the experiment, leading to an approximate discrepancy of 0.1 V in the band gap obtained using the two means.

### 2.4. PFET Performance

**Figure 6:**
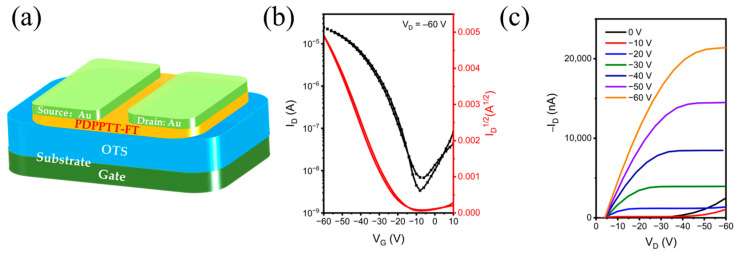
(**a**) The PFET device with BG-TC architectures; (**b**) transport characteristics and (**c**) output characteristics of PFET based on material of PDPPTT-FT.

We fabricated a bottom-gate/top-contact (BG/TC)-structured PFET device based on a π-conjugated polymer of PDPPTT-FT to characterize the charge transport behavior of the materials, and the corresponding device configuration is shown in Figure 6a. Figure 6b,c and Table 2 display and summarize the characteristic transfer and output plots of the transistors as well as the device performance. The annealed polymer shows excellent p-type transport properties with a maximum saturation mobility of 0.30 cm^2^ V^−1^ s^−1^. The average carrier mobility is in the vicinity of 0.25 cm^2^ V^−1^ s^−1^, which meets the performance requirements of a wide range of organic electronic devices, such as chemical sensors, radio frequency identification tags, thermoelectrics, etc. [40,41]. The threshold voltage (V_th_) and switching ratio (I_ON_/I_OFF_) of the device are –11 V and 10^4^, respectively, showing the low start-up characteristics and high sensitivity of the material.

### 2.5. Thin-Film Morphology

**Figure 7:**
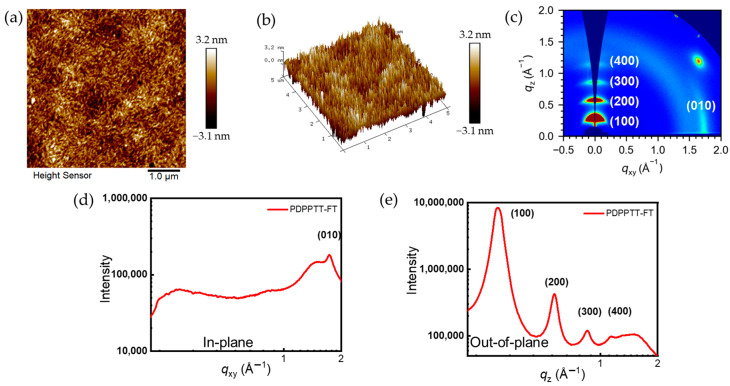
(**a**,**b**) AFM height and three-dimensional image; (**c**) thin film 2D-GIWAXS and (**d**,**e**) 1D-GIWAXS of PDPPTT-FT.

Thin-film microstructures, including surface morphology and molecular stacking patterns, play an important role in the charge transport properties of polymer semiconductors. Therefore, the microstructures of the films were characterized via scanning atomic force microscopy (AFM) and two-dimensional grazing incidence wide-angle X-ray scanning (2D-GIWAXS), which were performed under the same conditions as those used to fabricate PFET devices. The annealed film shows a fibrous intercalation network and distinct crystalline regions with a root mean square (RMS) of 1.02 nm. The high surface roughness of the annealed film may be associated with the good crystallinity. The 2D-GIWAXS diffraction patterns of the annealed films are collected and presented in Figure 7. The annealed polymer film shows arc-shaped four-level (h00) Bragg peaks (distinct multiple peaks: (100), (200), (300) and (400)) in the out-of-plane direction (q_z_ axis). In addition, the Bragg peaks of (010) accompanied by (100) of the film are located in the in-plane direction (q_xy_ axis), which suggests that the PDPPTT-FT adopts an edge-on stacking-dominated mode accompanied by face-on stacking in the thin-film state [42,43]. Edge-on facilitates efficient charge transport between chains, whereas the face-on stacking modes modulate the carrier injection barrier. The combination of the two modes favors the promotion of increased mobility [44]. The π–π stacking distances and the respective d–d stacking distances of PDPPTT-FT are 3.63 Å and 21.66 Å, respectively, as estimated from the values of the (010) and (100) signals in the q_xy_ and q_z_ directions (Table 3) [45]. The shorter π–π stacking distance of the material is believed to enhance the charge–carrier hopping between molecular chains and contribute to the hole mobility. Figure 7d,e also shows the 1D diffraction patterns and the corresponding calculated data.

## 3. Materials and Methods

Materials: The precursor DPPTT used for the synthesis was prepared using similar synthetic conditions as previously reported [46]. FT-Sn was purchased from SunaTech (Suzhou, China).

Synthesis of PDPPTT-FT polymer: DPPTT (200.00 mg, 0.17 mmol, 1.0 equiv.), FT-Sn (93.30 mg, 0.17 mmol, 1.0 equiv.) and tris(o-tolyl)phosphine (P(o-tol)_3_, 3.62 mg, 14.14 µmol, 8%) were weighed in a Schlenk tube. Afterward, dry chlorobenzene (12 mL) was added to the system. Argon was passed into the tube for 15 min to completely remove oxygen out of the system. Subsequently, tris(dibenzylideneacetone)dipalladium ([Pd_2_(dba)_3_], 3.25 mg, 3.53 µmol, 2%) was added. The polymerization process was continuously carried out at 130 °C with stirring for three days and the color of the mixture changed from orange–red to dark green. The purification process of the polymer was carried out via Soxhlet extraction. The oligomers were sequentially extracted with methanol, hexane, ethyl acetate and acetone under reflux conditions for 12 h each. The target product was extracted using chloroform. The fractions were then added dropwise to methanol (180 mL) and pump-filtered to afford a dark green powdered solid (83% yield).

The device preparation steps for the PFETs are presented in the Supplementary Materials.

## 4. Conclusions

In this work, π-conjugated polymer PDPPTT-FT with high molecular weight was prepared via rational design using Stille polymerization. This material has good solubility and thermodynamic stability, providing processability for the subsequent application of the material used in transistors. The F...S interaction force between atoms favor the formation of intramolecular conformational locks, thereby maintaining the planarity of the structure. PFET devices prepared with PDPPTT-FT as an organic semiconductor show maximum and average hole mobilities of 0.30 cm^2^ V^−1^ s^−1^ and 0.25 cm^2^ V^−1^ s^−1^, which we attribute to the coplanarity and high crystallinity of the material. Our laboratory is currently investigating the functionalization of this polymeric material for stretchable and self-healing applications.

## Figures and Tables

**Table 1:** Test results for molecular weight and elemental content of PDPPTT-FT.

Molecule	Mn	Mw	PDI	C	H	N
				(%)	(%)	(%)
PDPPTT-FT	23,596	88,133	3.73	71.50 ^1^	8.63 ^1^	2.02 ^1^
repeating unit ^2^	N/A	N/A	N/A	71.62	9.10	2.39

^1^ Mean value of two tests; ^2^ Calculated theoretical values.

**Table 2:** Carrier performances of PDPPTT-FT-based PFET device.

	Coating Speed (rpm)	Annealing Temperature (°C)	Carrier Type	Max Mobilities (cm^2^/(V s))	Average Mobilities ^1^ (cm^2^/(V s))	V_th_ (V)	I_ON_/I_OFF_
PDPPTT-FT	2000	180	Hole	0.30	0.25 ± 0.05	−11	10^4^

^1^ Data were obtained from 10 devices.

**Table 3:** The crystallographic information of the polymer thin film.

Material	In-Plane (010) Peak (Å^−1^)	π-Spacing (Å) ^1^	In-Plane (100) Peak (Å^−1^)	d-Spacing (Å) ^1^	Out-of-Plane (100) Peak (Å^−1^)	d-Spacing (Å) ^1^
PDPPTT-FT	1.73	3.63	0.27	23.26	0.29	21.66

^1^ Calculated via the Bragg’s Equation 2dsinθ = nλ.

## Data Availability

The data presented in this study are available in article and Supplementary Materials.

## Supplementary Materials

The following supporting information can be downloaded at: https://www.mdpi.com/article/10.3390/molecules29010260/s1, Figure S1: Gel permeation chromatography data test for the polymer; Figure S2: HOMO−1, HOMO−2, LUMO+1 and LUMO+2 map of the dimer of PDPPTT-FT; Table S1: The spatial atomic coordinates of the dimer.
