# Supplementary material for: Incorporation of Diketopyrrolopyrrole into Polythiophene for the Preparation of Organic Polymer Transistors
Source: Molecules. 2024 Jan 3;29(1):260. doi: 10.3390/molecules29010260 (PMC10780697; doi:10.3390/molecules29010260)
Supplement: Supplementary file 1 [file molecules-29-00260-s001.zip › molecules-2800573-supplementary.pdf]

Supplementary Information

Figure S1. Gel permeation chromatography data test for the polymer.

Calibration Type: Narrow Standard Curve Fit Used: 1 K: 14.1000 Alpha: 0.7000
Calibration Curve: y = 12.502019 - 0.602947x^1
High Limit MW RT: 9.88 mins Low Limit MW RT: 17.07 mins
Flow Marker RT: 0.00 mins FRCF: 1.0000 FRM Name:

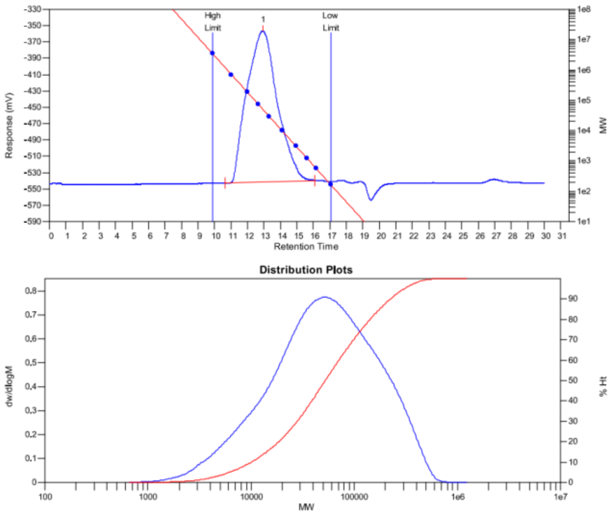

MW Averages

Peak No	Mp	Mn	Mw	Mz	Mz+1	Mv	PD
1	51754	23596	88133	196225	295226	75479	3.73508

Processed Peaks

Peak No	Name	Start RT (mins)	Max RT (mins)	End RT (mins)	Pk Height (mV)	% Height	Area (mV.secs)	% Area
1		10.63	12.92	16.07	184.96	0	23829.4	100

The molecular weight of the macromolecules was evaluated by gel permeation chromatography (eluent: trichlorobenzene, 150°C). The degree of dispersion of the PDPPTT-FT = Mw/Mn.

Figure S2. HOMO−1, HOMO−2, LUMO+1 and LUMO+2 map of the dimer of PDPPTT-FT.

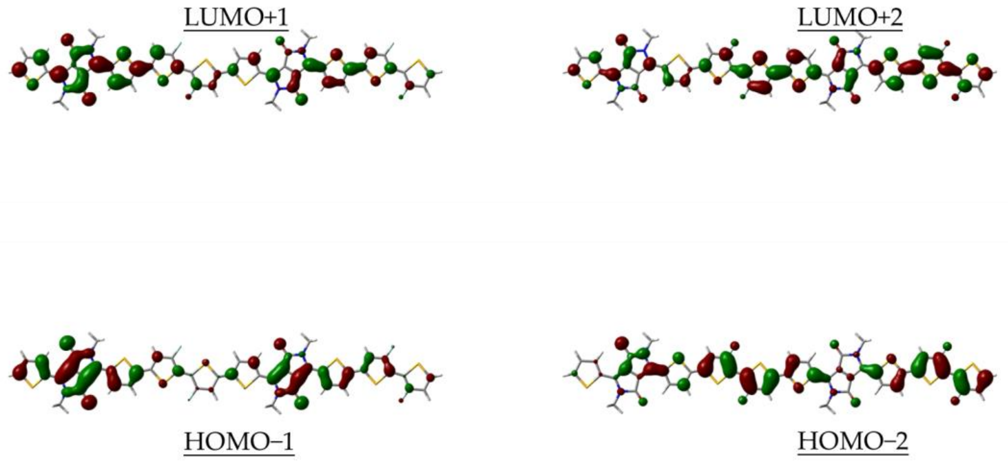

Energy levle characterization: Thermal stability measurements were conducted at 30-650 °C under nitrogen. Electrochemical tests were carried out in acetonitrile solution containing tetrabutylammonium hexafluorophosphate under argon. The Ag/AgCl electrode, glassy carbon electrodes and platinum electrodes were applied as reference electrodes, working electrodes and counter electrodes, respectively. 3 μL of a chlorobenzene solution of the polymer at a concentration of 0.5 mg/mL was dispensed with a pipette gun and dropped onto the glassy carbon electrode, which was allowed to evaporate with the aid of pressure from the washout bulb at room temperature (the electrode's circular hole had a diameter of 4 mm).

PFET device: Highly doped n-type silicon (Si) wafers with a silicon dioxide (SiO_2) content of 300 nm were used as substrates, which were ultrasonically cleaned in deionized water, acetone and isopropanol for 5 min, respectively, and then processed in the UV-zone for 30 min. After that, the substrate was modified with octadecyltrichlorosilane (OTS). For the preparation of semiconductor films, the PDPPTT-FT are pre-dissolved in chlorobenzene (5mg/ml) and heated overnight at 80 °C with stirring. Subsequently, the polymer solution was spin-coated onto OTS-treated Si wafers at 2000 rpm for 60 s, and then annealed at 180 °C for 30 min in a glove box (nitrogen atmosphere). Finally, source-drain electrodes of Au were deposited by evaporation in vacuum (W/L = 11, the channel width to length ratio) to accomplish device preparation. OFET characterizations were carried out in glove box using the 4200-semiconductor system. The saturated hole mobility (μ) is calculated as: $\mu = (\frac{\partial\sqrt{|I_{DS}|}}{\partial V_{GS}})^2 \cdot \frac{2L}{WCi}$. In which I_{DS} and V_{GS} are the source-drain current and gate voltage, respectively; Ci is the capacitance per unit area of the gate dielectric layer.

Table S1: The spatial atomic coordinates of the dimer of PDPPTT-FT.

C	-15.83682300	-0.48269900	-0.00138300
C	-14.76064600	0.40930200	0.00112900
C	-13.53801300	-0.32361700	-0.00113800
C	-13.84804600	-1.72662800	-0.00523900
N	-15.28824300	-1.77305200	-0.00524700
C	-14.45157800	1.81273100	0.00521600
N	-13.01209200	1.85987500	0.00523200
C	-12.46135300	0.56827800	0.00139600
O	-13.13856700	-2.76015000	-0.00833800
O	-15.16079700	2.84633300	0.00827600
C	-11.07655800	0.23269500	0.00035000
C	-17.22668700	-0.14776700	-0.00017000
C	-15.97121100	-3.06116000	-0.00900300
C	-12.32939600	3.14778600	0.00908500
S	-18.59303800	-1.36876900	-0.00327200
C	-19.77499700	-0.02101600	0.00081300
C	-19.16196200	1.19814900	0.00408700
C	-17.74082700	1.13543100	0.00356300
S	-9.71307900	1.45339400	0.00362700
C	-8.50435600	0.09460500	-0.00052700
C	-9.15274500	-1.12492500	-0.00380400

C	-10.56012200	-1.05420300	-0.00333400
C	-7.11005300	0.38146100	-0.00010800
C	-6.46257500	1.59693300	0.00072000
C	-5.06237200	1.47535500	0.00083800
C	-4.52392200	0.21014800	0.00010000
S	-5.90076600	-0.99364100	-0.00066200
C	-3.16534700	-0.19580900	-0.00009800
S	-1.78903000	1.00828600	0.00044200
C	-0.57917600	-0.36651600	-0.00041800
C	-1.22649000	-1.58223600	-0.00088800
C	-2.62660600	-1.46103900	-0.00067800
C	0.81457800	-0.07899600	-0.00050300
C	1.46193500	1.14163700	-0.00242100
C	2.86872400	1.07267900	-0.00203100
C	3.38718900	-0.21418200	0.00022000
S	2.02468600	-1.43654200	0.00213500
F	-4.24809700	2.60861500	0.00153800
F	-3.44065800	-2.59444500	-0.00119000
C	4.77119400	-0.54741800	0.00088800
C	5.84798100	0.34705300	-0.00070200
C	7.06977300	-0.38303000	0.00069800
C	6.76307600	-1.78747300	0.00327900

N	5.32467800	-1.83771900	0.00328900
C	6.15504100	1.75165200	-0.00329900
N	7.59335500	1.80182800	-0.00335900
C	8.14669300	0.51159900	-0.00095200
O	7.47607800	-2.81868200	0.00524600
C	4.64426100	-3.12705900	0.00581400
O	5.44208300	2.78278700	-0.00521100
C	9.53081800	0.17876600	-0.00031800
C	8.27391900	3.09114400	-0.00568100
S	10.89336700	1.40151400	-0.00280100
C	12.10328200	0.04459100	-0.00029300
C	11.45705500	-1.17586000	0.00218000
C	10.04958500	-1.10753500	0.00217400
C	13.49827600	0.33379100	-0.00104000
C	14.14407800	1.54827400	-0.00451900
C	15.54685000	1.42672700	-0.00412600
C	16.08358400	0.16394500	-0.00049900
S	14.70865900	-1.03982300	0.00299500
C	17.44631800	-0.24867800	0.00066800
S	18.82939200	0.94927300	-0.00282900
C	20.00620600	-0.41761900	0.00108900
C	19.39057900	-1.63074000	0.00460900

C	17.97673400	-1.51249000	0.00430400
F	16.35806400	2.56250400	-0.00740600
F	17.16425900	-2.64753400	0.00760200
H	-15.19096100	-3.81823500	-0.01140600
H	-16.58596600	-3.18773400	0.88073200
H	-16.58625000	-3.18237600	-0.89928900
H	-11.71535200	3.27060400	0.90005800
H	-11.71562000	3.27609300	-0.88130200
H	-13.11031400	3.90437200	0.01152000
H	-20.82408800	-0.25534800	0.00039200
H	-19.70974100	2.12767500	0.00686600
H	-17.09508800	2.00345000	0.00584900
H	-8.61585100	-2.06154900	-0.00667300
H	-11.20858900	-1.92033000	-0.00576200
H	-6.96392200	2.55111200	0.00105000
H	-0.72496200	-2.53634000	-0.00155000
H	0.92381900	2.07753600	-0.00423300
H	3.51621700	1.93958200	-0.00349000
H	5.42620600	-3.88252400	0.00744300
H	4.03048800	-3.25150400	0.89668800
H	4.03072500	-3.25513600	-0.88471300
H	8.88771700	3.21871700	0.88470300

H	8.88759700	3.21558000	-0.89659600
H	7.49217500	3.84679000	-0.00697100
H	11.99575500	-2.11137600	0.00390900
H	9.40253400	-1.97480400	0.00386700
H	13.64321900	2.50275500	-0.00736800
H	21.05641700	-0.19273500	0.00041600
H	19.89918600	-2.58065200	0.00734200
